# The dimensional and morphological assessment of the frontal sinus in sex estimation among different populations

**DOI:** 10.1186/s13005-023-00355-4

**Published:** 2023-03-14

**Authors:** Bowen Zheng, Yuan Zhong, Nassem Ali Al-Worafi, Yi Liu

**Affiliations:** Department of Orthodontics, School and Hospital of Stomatology, China Medical University, Nanjing North Street 117#, He-Ping District, Shenyang, 110002 P. R. China

**Keywords:** Bosnian, Chinese, Frontal sinus, Lateral cephalometric, Nepalese

## Abstract

**Background:**

The purpose of the present study was to determine the dimorphic potential of the frontal sinus (FS) index, area, and the combination of both variables to ascertain its accuracy in sex discrimination among Bosnian, Chinese, and Nepalese.

**Methods:**

A retrospective study analyzed the digital standardized lateral cephalometric of 654 Bosnian, Chinese, and Nepalese adult patients (116 females, 102 males; age range 17–40 years). The following FS parameters were analyzed: The maximum height, width, and area of the FS, and the ratio of height and width was calculated as the FS index. The measurements were analyzed using logistic regression analysis for the FS index, area, and combined FS index and area.

**Results:**

Statistically significant differences were observed between the mean values of the FS index and the area between females and males in each population. The highest frequency of bilateral absence of FS was detected among females, specifically in Chinese (6.6%). Logistic regression equation derived from the present study differentiated between sexes with higher classification percentages for the FS index and area with 62.4%, 75.2%, and 78.4% among Bosnian, Chinese, and Nepalese subjects, respectively.

**Conclusions:**

The results of the present study highlight the implications of the combined FS index and area as a reliable approach in sex estimation in forensic science whenever both the FS structure and lateral cephalometric are available.

## Introduction

Sex estimation is a key analysis that forensic anthropologists perform to establish a biological profile of individual remains [1]. The skull is usually regarded as the most reliable indicator for sex discrimination after the pelvis [2, 3]. Brow ridge shape, nasal aperture size [1], or mastoid process may contribute to sex estimation when studying the anatomical skull features [2]. However, these may be inapplicable in subjects with fractured and deformed skulls. Thus, finding an alternate sexually dimorphic diagnostic feature is necessary [2].

The frontal sinuses (FSs) cavities have great variability and are located inside the frontal bone that originates from ethmoidal cells [4, 5]. The FSs are unique paired [4], irregular lobulated cavity structures that lie posterior to the superciliary arches [6–10]. The FS is not affected by the time elapsed until post-mortem and is considerably less involved by external factors (except by the rare occurrence of fractures, tumors, or some severe infections) [11, 12]. The FS is so distinctive and similar to fingerprints that the opportunity of two individuals having identical morphology of the FS is extremely remote [13]. Therefore, FS properties make it a suitable indicator for sex estimation in forensic studies [2].

In the absence of fingerprints and DNA samples, radiographic recognition plays an essential role in forensic medicine [14, 15]. Following the establishment of lateral cephalograms in the orthodontic field by Broadbent in 1931 [16], the utilization of this technology for legal purposes has progressively expanded [7]. The FS component of the craniofacial structure is detectable in the lateral cephalogram because of its unique cavity structure [17]. Thus, it has been demonstrated that the FS constituted a reference scale for measurement and significantly improved the reliability of sex estimation [16, 18, 19].

Numerous previous studies have researched the potential of the FS measurements to determine sexual dimorphism among Chinese [2], Indian [10, 20, 21], Nigerians [22], and French [23] populations using Two-dimension (2D) [2, 10, 20–22], and Three-dimension (3D) [23] radiographic tools, respectively. With different approaches, such as the FS index technique (the ratio of the maximum height to the depth of frontal sinus) and area. The results of those investigations indicated that the measured FS index technique has potential implications for sex classification [2, 10, 22]. However, a recent investigation revealed that the proper indicator was the combined FS index and area, stating that the FS index alone reduces the correct estimation rate [2].

To date, the assessment of FS index, area, and combined variable among different racial groups has not been investigated. Therefore, the present study aimed to assess the validity of sex classification accuracy using the FS index, area, and combined FS index and area in forensic investigations in Bosnian, Chinese, and Nepalese subjects based on 2D radiographic examination. The authors hypothesized that regarding sex estimation, there would be no difference in the FS index, area, and combined variables.

## Materials and Methods

This study was approved by the Local Research Ethics Committee of Stomatology of China Medical University in Shenyang following the Declaration of Helsinki. A retrospective study was designed, and data were collected from patient data from the Department of Orthodontics, Stomatology Hospital, China Medical University in Shenyang, China for Chinese subjects. At the same time, Caucasians and Asians from Nepal subjects were collected electronically via email [24–26] (Table 1). In total, 744 digital lateral cephalometric radiographs were examined, which were obtained from March 2016 to November 2018. Some measurement values were rejected (32 samples) due to exclusion criteria. Fifty-eight samples of 712 were also excluded due to the bilateral absence of the FS. The final sample consisted of 654 lateral cephalometric radiographs (218 in each group, 116 female and 102 male aged from17-40 years (Table 2). Lateral cephalometric radiographs of subjects presenting normal anatomical features and good image quality of FS were included in the sample. Radiographs with surgical intervention; apparent pathology in FS; abnormal enlargement of FS; suffered a head collision; multi-reagent chemotherapy; craniofacial syndromes; trauma; cleft lip and palate were excluded [2, 20, 24].

**Table 1:** Study data collection and radiographic instrumentation

	**Bosnia and Herzegovina**	**China**	**Nepal**
City	Banja Luka	Shenyang	Dhulikhel
Population	Bosnian	Chinese	Nepalese
Subjects, *n*	218	218	218
Data collection site	Private clinic	Department of Orthodontics, School of Stomatology, China Medical University	Private clinic
Radiographs model	Planmeca ProMax® 3D Max	Proline XC2009	Vatech, Pax- i3D Smart™
kVp	82	64 to 68	50 to 90
mA	13–15	5 to 6	4 to 16
Exposure time/s	12.1	12.8	12.9
Software	Planmeca Romexis software	Planmeca Dimaxis Pro/Classic 4.2.0 version,	Vatech EzDent Software V4
Origin	Helsinki, Finland	Helsinki, Finland	Korea

**Table 2:** The mean age of study groups in female and male in each population

Variable	Age (17–40 years)			
	Female (116)		Male (102)	
	Mean	SD	Mean	SD
Bosnian	17.81	7.828	18.76	4.959
Chinese	18.61	8.102	18.26	8.491
Nepalese	17.1	8.004	18.32	6.556
**The percentage of bilateral absence of frontal sinus**				
**Bosnian (*n* = 231)**	7 (3%)		6 (2.6)	
**Chinese (*n* = 244)**	16 (6.6%)		10 (4.1)	
**Nepalese (*n* = 237)**	13 (5.5%)		6 (2.5%)	

Note: Age group of each population presented as mean ± standard deviation (SD)

All 2D radiographs were evaluated and measured by the principal author. The dimensions of the FS were measured using a digitizing tool WinCeph version 8.0 software (Rise Corporation, Sendai, Japan), based on the technique described by the previous literature [2]. The imaging analysis software was calibrated to account for any variations in magnification due to the radiographic instrument and use of the cephalostat. One observer measured the area, maximum height, and width of the FS in centimeters.

**Fig. 1:**
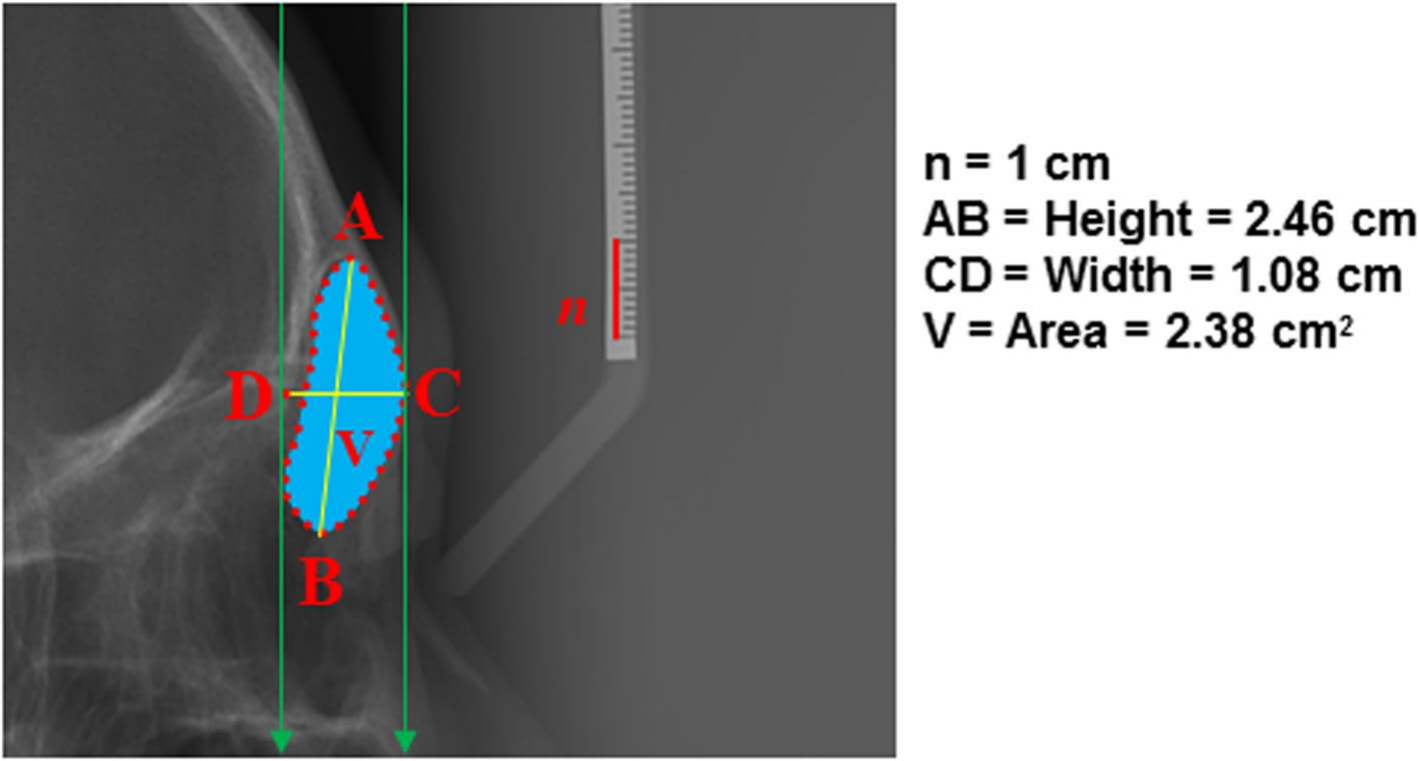
Lateral cephalometric measurements of the frontal sinus. (A) the highest point, (B) the lowest point, (AB) indicates the maximum height of the frontal sinus, (C) anterior point on the wall of the frontal sinus, (D) posterior point of the wall of the frontal sinus, (CD) indicates the maximum width of the frontal sinus. The rate of the height (AB) and width (CD) was calculated as frontal sinus index

First, to measure the area, the outline of the FS was traced, then calculated directly from the software. Second, to determine the FS index, the highest landmark (A) was noted on the FS and connected to the lowest landmark (B) to obtain the maximum height of FS (AB). Likewise, the maximum width of FS was determined by connecting landmark C (anterior wall of the FS) to landmark D (posterior wall of the FS), which is perpendicular to line AB (Fig. 1). To determine the FS index [2, 10], the ratio of AB and CD was calculated using the Microsoft Excel (Microsoft, Redmond, Washington, USA) through the following formula: FS index = AB/CD.

### Reliability of measurements

To reduce potential bias due to intra-examiner variability, 20 lateral cephalometric radiographs were randomly selected from each racial group and re-measured by the same evaluator one month following the initial analysis. In this context, the intra-class correlation coefficient (ICC) was applied. The ICC estimates and the 95% confidence intervals were calculated based on a mean-rating, consistency, two-way mixed-effects model. The ICC is a broadly practised index for measurement reliability to evaluate the reproducibility of the readings [24, 27].

### Statistical analysis

Data were assessed using IBM SPSS Statistic software (version 26 IBM SPSS Inc., Chicago, IL, USA). Intra-class correlation coefficient (ICC) test was applied to measure the Intra- observer agreement with 95% confidence intervals. Descriptive statistics were generated for all variables, such as mean values and standard deviations. The Kolmogorov–Smirnov normality test was applied to check the normal distribution of sex and the FS values (FS index and area). Non-parametric Kruskal–Wallis H test was applied in conjunction with an unpaired Mann–Whitney U test for non-normally distributed variables. Logistic regression analysis was applied, sex was carried out as a dependent variable, and the FS index and FS area separately and as a combination were used as the covariate variables with the confidence of intervals of 95%. A *p*-value < 0.05 was considered significant.

### Results

The results of reliability statistics for the frontal sinus area and AB and DC dimensions for Bosnian were (0.96, 0.92, and 0.96), Chinese (0.94, 0.92, and 0.96), and Nepalese (0.92, 0.98, and 0.92), respectively. These results imply a high degree of reliability of the re-measured radiographs. Based on the 95% confidence interval of the ICC estimate, values of > 0.90 indicated excellent reliability [27].

The highest frequency of bilateral absence of the FS was recorded in China, followed by Nepal and Caucasians, specifically among females with 6.6%, 5.5%, and 3%, respectively. The identical frequency of the FS absence was observed among Bosnian and Nepalese male cases were 2.6%. Nonetheless, the absence of the FS occurred in Chinese males with the highest frequency of 4.1% (Table 2).

The mean index was larger in the female than the male groups in all populations. The FS index differences present significant differences between males and females in Chinese and Nepalese groups. The outcomes of the FS area revealed that the value among males was larger than females in Bosnian, Chinese, and Nepalese, with a statistically significant difference (*p* < 0.01), as presented in Table 3. In Table 4, the FS index presented the lowest value for sex classification (56.9%, 67%, and 62.8%).

**Table 3:** Mean measurements of frontal sinus index and area in females and males

**Variable**		**Sex**	**Mean**	**SD**	**t-test**	***P* value**
**Bosnian**	Index	Female	2.80	0.545	1.97	0.050
		Male	2.49	0.564		
	Area	Female	1.85	0.824	2.01*	0.047
		Male	2.32	0.917		
**Chinese**	Index	Female	2.98	0.532	2.02*	0.043
		Male	2.50	0.254		
	Area	Female	1.97	0.587	2.13*	0.030
		Male	2.50	0.853		
**Nepalese**	Index	Female	2.55	1.083	1.99*	0.049
		Male	2.13	0.460		
	Area	Female	1.67	0.556	2.99**	0.011
		Male	2.61	0.839		

Note: Number of subjects (Male = 102; Female = 116), Mann–Whitney U test was used, *SD* standard deviation, all measured values were in centimetres ** *p* < 0.01, * *p* < 0.05

**Table 4:** Frontal sinus index classification results from the logistic regression analysis

	**Sex**	**Female**	**Male**	**Total**	**Classification%**
**Bosnian**	Female	68	48	116	58.6
	Male	46	56	102	54.9
	Total	114	104	118	56.9
	Equation	L = -1.059 Index + 2.664			
**Chinese**	Female	81	35	116	69.8
	Male	37	65	102	63.7
	Total	118	100	218	67.0
	Equation	L = -1.211 Index + 3.255			
**Nepalese**	Female	82	34	116	70.7
	Male	47	55	102	53.9
	Total	129	89	218	62.8
	Equation	L = -1.318 Index + 2.907			

*L* Logistic regression equation

The FS area yielded a sex allocation accuracy of (60.6%, 69.3%, and 76.1%) when each variable was carried out as a single classifier in Bosnian, Chinese, and Nepalese subjects, respectively (Table 5). Table 6 indicates that the combined FS index and area approach for classified sex with 62.4%, 75.2%, and 78.4% in Bosnian, Chinese, and Nepalese subjects, respectively, is effective and provides more accurate predictions in sex estimation as supported by logistic regression analysis.

**Table 5:** Frontal sinus area classification results of the logistic regression analysis

	**Sex**	**Female**	**Male**	**Total**	**Classification%**
**Bosnian**	Female	84	32	116	72.4
	Male	54	48	102	47.1
	Total	138	80	218	60.6
	Equation	L = 0.629 Area – 1.432			
**Chinese**	Female	89	27	116	76.7
	Male	40	62	102	60.8
	Total	129	89	218	69.3
	Equation	L = 1.523 Area -3.550			
**Nepalese**	Female	91	25	116	78.4
	Male	27	75	102	73.5
	Total	118	100	218	76.1
	Equation	L = 2.098 Area—5.078			

*L* Logistic regression analysis

**Table 6:** Frontal sinus index and area classification results of the logistic regression analysis

	**Sex**	**Female**	**Male**	**Total**	**Classification%**
**Bosnian**	Female	79	37	116	68.1
	Male	45	57	102	55.9
	Total	124	94	218	62.4
	Equation	L = -1.124 Index + 0.688 Area + 1.409			
**Chinese**	Female	91	25	116	78.4
	Male	29	73	102	71.6
	Total	120	98	218	75.2
	Equation	L =—1.136 Index + 1.503 Area—0.316			
**Nepalese**	Female	95	21	116	81.9
	Male	26	76	102	74.5
	Total	121	97	218	78.4
	Equation	L =—1.312 Index + 2.098 Area—2.012			

*L* logistic regression equation

When the present study was compared to the current international data [2, 10, 20–23], it was found that the correct estimation was in the range of 60% to 76.7%, even with the various subject number and radiographic techniques used as listed in Table 7.

**Table 7:** The results of the frontal sinus in sex estimation in Bosnian, Chinese, and Nepalese with current worldwide data

References	Country	City	Number of subjects	Correct Discrimination classification %	Radiographic Instrument
Present study	Bosnia and Herzegovina	Banja Luka	218	62.4	Lateral cephalometric
	China	Shenyang	218	75.2	
	Nepal	Dhulikhel	218	78.4	
Luo et al. [2]	China	Xinjiang	475	76.7	Lateral cephalometric
Eboh et al. [23]	France	Marseille	69	72.5	Computed tomography
Goyal et al. [21]	India	Belgaum	300	64.6	Postero-anterior radiograph
Michel et al. [20]	India	Sattur	100	60	Paranasal sinus (PNS)-view radiographs
Ramaswamy et al. [10]	India	Eluru	216	67.59	lateral cephalometric radiographs
Belaldavar et al. [22]	Nigeria	Abraka	216	60	Postero-anterior radiographs

## Discussion

In this retrospective study, the FS index, area, and combined variables were investigated to establish whether scientifically sound sex estimation was possible among three populations. With the advent of new cutting-edge technologies in radiology, obtaining accurate and reliable measurements has become routine [2]. Lateral cephalometric is commonly used in dentistry, specifically by orthodontists, to diagnose, plan treatment, trace, and during orthognathic or implant surgery[7, 28]. The establishment of sex estimation by measuring 2D radiographs is deemed preferable to identification by photographic superimposition since radiographic evaluation is less time-consuming and technically exacting [28, 29].

The rationale for selecting these age groups was that the growth of this anatomical structure is slow [30], and the FSs complete their development and reach their maximum size at the age of 15 or 20 years [19, 23, 28, 30] and then remain stable until death [23, 31]. However, some of the radiographs could not be measured either because no FS was visible or because the radiograph did not meet the inclusion criteria. The radiographic diagnosis of the FS was performed according to previously reported literature that has identified the bilateral absence of FS [2]. The results of the present study revealed that the frequency of bilateral missing FS was identical in males among Bosnian and Nepalese (2.8%). It seems that the percentage of FS absences in females and males is higher in Chinese than in Bosnian and Nepalese subjects. Luo et al. [2] found that the frequency of bilateral FS absence was 9% for females and 5% for males. A greater frequency of bilateral absence was observed among females than males [30], which is identical to the observation of the present study. However, bilateral absence of FS was not observed in Manisa in Turkey among 100 cases when using paranasal computed tomography (CT) scans for identifying unknown bodies [11] and in 69 patients in Marseille in France for sex determination using 3D reconstructions [23]. According to the literature, bilateral absence of the FS is reportedly the least frequent, and the FS is present in 90% of adults[30].

It is important to emphasize that the FS index[2, 10] and FS area [2] have shown relatively higher accuracy for sex estimation in 2D radiographs [2, 10]. The rationale for using the FS index [10] and area was based on previously established criteria [2]. The methods of sex estimation using the FS index and FS area could be carried out due to their relative simplicity and short processing time, allowing for precise and reliable results [32, 33].

This study observed a high degree of variability between the individuals in each population group. Significant differences were observed in the study groups' FS index and FS area measurements between females and males. This result is in line with previously reported studies depicting a statistically significant sexual dimorphism of the FS index [10] and area [2]. The current outcome supports the dimorphic features of FS that have implications for individual identification [10, 22]. Various investigators have generally targeted the FS index [2, 10, 20–23] and both variables [2] to achieve sex estimation in their own population.

In this study, identical approaches were adopted in various population groups. The results indicated that the application of the FS index revealed the lowest sex estimation percentage compared to the proposed use of FS area and both variables with a difference of 3.7%, 2.3%, and 13.3% to FS area and with a difference of 5.5%, 8.2%, and 15.6% to both variables in Bosnian, Chinese, and Nepalese respectively. A study in India applied the FS index for sex estimation using 300 digital posteroanterior radiographs and achieved 64.6% sex discrimination with the logistic regression analysis [21]. A 2D radiograph was used among 216 Indian subjects, and the estimation function equation was used for the correlation between sex and FS index and achieved 67.6% correct sex estimation [10]. In this study, the outcomes of the FS index were lower than in previous studies when this approach was adopted, presenting 56.9%, 67.0%, and 62.8% in sex differentiation among Bosnian, Chinese, and Nepalese, respectively.

The results present the first attempt to estimate sex differentiation from the FS area. It was observed that the use of the FS area exhibits a lower percentage of correct sex estimation percentage than the application of both FS index and area, with a difference of 1.8%, 5.9%, and 2.3% in Bosnian, Chinese, and Nepalese, respectively. There is no previous supportive study to show the application of the FS area presents a higher percentage classification rate than the FS index. A study in France measured the total volume of the left and right FS with the application of 3D reconstruction of FS to determine sexual dimorphism among 69 cases and achieved 72.5% [23]. Although the results of the FS area indicated lower differences compared to the FS index, applying the FS index and area together is recommended based on 2D radiographic assessment [2]. Previous literature has improved the prediction model by combining FS index and area, which commit an overall sex classification accuracy of 76.6% among 475 cases in China [2]. It was found in a current study that, after being subjected to using FS index and area, the correct sex classification rate was 62.4%, 75.2%, and 78.4% in Bosnian, Chinese, and Nepalese, respectively. The use of the FS index and area presented the highest correct estimation percentage (Table 7), which implied that these variables were distinctly and quantifiably different at a high level and created a proper indicator, and promoted the discrimination rate compared to those in the other studies used merely the FS index [2]. The current findings highlighted a potential influence of the FS index and area on sex estimation, which rejected the hypothesis.

The current study compared outcomes with the current global datasets regarding sex estimation from the FS (Table 7) [2, 10, 20, 21, 23]. The correct estimation percentage in Caucasians (62.4%) in this study was lower than those studies conducted in China [2] (76.7%), France [23] (72.5%), and India (67.59% [10], 64.6%[21]). A previous study was conducted to determine the FS's dimorphic potential using a logistic regression model among 216 posterior-anterior Nigerian radiographs. It was concluded that the left-side FS width presented the highest accuracy of 60% in sex determination [22]. However, in the present study, China and Nepal demonstrated greater correct sex estimation with 75.2% and 78.4%, respectively. This discrepancy can be attributed to factors such as ethnicity, the use of various landmarks, the degree of radiographic enlargement, and techniques [24]. The remaining 37.6% Bosnian, 24.8% Chinese, and 21.6% Nepalese were not classified correctly. This may be attributed to high inter-individual variability in the morphology of the FS [10].

Assessing the FS index and area in other populations might be helpful, and familiarity with the FS values is relevant for successful surgery and anthropology [22]. Accurate selection of a reliable identification approach and appropriate measurement tools will assist in achieving reliable results. Applying the FS index and area technique can be considered an alternative identification modality to other procedures in sex estimation [2]. This highly versatile yet practical procedure allows forensic medicine and clinicians to rely on a simple, technique-insensitive, cost-effective material processing method.

Further research is required to explore and identify the difference in the FS of diseased persons between populations and factors that influence change in the FS that affects the decision of sex identification. The generalizability of these results is subject to certain limitations. For instance, 2D radiographs were selected in this study to measure FS and evaluate sex estimation, and this is mainly due to the high costs of CT examinations [28]. The limited number of lateral cephalometric studied in each population group is also a potential limiting factor.

The current study emphasized the implication of the FS as a positive tool in sex estimation through 2D radiographic assessment among various population groups. The bivariate models have borderline accuracy in the Nepalese population (just below 80% limit) while in the Chinese and Bosnian populations, the models are not recommended for use if there are alternative measurements in the cranium.

## Data Availability

The authors declare that the materials are available.

## Abbreviations

FS: Frontal sinus
2D: Two-dimension
3D: Three-dimension
ICC: Intra-class correlation coefficient

## References

[1] Williams BA, Rogers TL (2006). Evaluating the accuracy and precision of cranial morphological traits for sex determination. J Forensic Sci.

[2] Luo H, Wang J, Zhang S, Mi C (2018). The application of frontal sinus index and frontal sinus area in sex estimation based on lateral cephalograms among Han nationality adults in Xinjiang. J Forensic Leg Med.

[3] Rogers TL (2005). Determining the sex of human remains through cranial morphology. J Forensic Sci.

[4] Quatrehomme G, Fronty P, Sapanet M (1996). Identification by frontal sinus pattern in forensic anthropology. Forensic Sci Int.

[5] Prossinger H (2001). Sexually dimorphic ontogenetic trajectories of frontal sinus cross sections. Coll Antropol.

[6] Reichs KJ (1993). Quantified comparison of frontal sinus patterns by means of computed tomography. Forensic Sci Int.

[7] Da Silva RF, Prado FB, Caputo IGC, et al. The forensic importance of frontal sinus radiographs. J Forensic Leg Med. 2009;16:18–23. 10.1016/j.jflm.2008.05.016 19061844

[8] Christensen AM. Testing the reliability of frontal sinuses in positive identification. J Forensic Sci. 2005;50:JFS2004145–5. 15830992

[9] de Andrade Quintanilha Ribeiro F, (2000). Standardized measurements of radiographic films of the frontal sinuses: an aid to identifying unknown persons. Ear Nose Throat J.

[10] Ramaswamy P, Khaitan T (2014). Frontal sinus index–A new tool for sex determination. J Forensic Radiol Imaging.

[11] Tatlisumak E, Ovali GY, Aslan A (2007). Identification of unknown bodies by using CT images of frontal sinus. Forensic Sci Int.

[12] Kim D, Lee U, Park S (2013). Identification using frontal sinus by three-dimensional reconstruction from computed tomography. J Forensic Sci.

[13] Nambiar P, Naidu MDK, Subramaniam K (1999). Anatomical variability of the frontal sinuses and their application in forensic identification. Clin Anat.

[14] Buyuk SK, Karaman A, Yasa Y (2017). Association between frontal sinus morphology and craniofacial parameters: A forensic view. J Forensic Leg Med.

[15] Kirk NJ, Wood RE, Goldstein M (2002). Skeletal identification using the frontal sinus region: a retrospective study of 39 cases. J Forensic Sci.

[16] Patil AA, Revankar A, v,  (2013). Reliability of the frontal sinus index as a maturity indicator. Indian J Dent Res.

[17] Tehranchi A, Motamedian SR, Saedi S (2017). Correlation between frontal sinus dimensions and cephalometric indices: A cross-sectional study. Eur J Dent.

[18] Choi IGG, Duailibi-Neto EF, Beaini TL (2018). The frontal sinus cavity exhibits sexual dimorphism in 3D cone-beam CT images and can be used for sex determination. J Forensic Sci.

[19] Beaini TL, Duailibi-Neto EF, Chilvarquer I, Melani RFH (2015). Human identification through frontal sinus 3D superimposition: Pilot study with Cone Beam Computer Tomography. J Forensic Leg Med.

[20] Michel J, Paganelli A, Varoquaux A (2015). Determination of Sex: Interest of Frontal Sinus 3 D Reconstructions. J Forensic Sci.

[21] Goyal M, Acharya AB, Sattur AP, Naikmasur VG (2013). Are frontal sinuses useful indicators of sex?. J Forensic Leg Med.

[22] Belaldavar C, Kotrashetti VS, Hallikerimath SR, Kale AD (2014). Assessment of frontal sinus dimensions to determine sexual dimorphism among Indian adults. J Forensic Dent Sci.

[23] Eboh DEO, Ogbeide OU, Ivwighren T (2017). Radiographic anthropometric study of frontal sinus for sex determination in Benin city. South-South Nigeria J Forensic Dent Sci.

[24] Muhammed FK, Abdullah AO, Rashid ZJ (2019). Morphology, incidence of bridging, and dimensions of sella turcica in different racial groups. Oral Radiol.

[25] Chang Z-C, Hu F-C, Lai E (2011). Landmark identification errors on cone-beam computed tomography-derived cephalograms and conventional digital cephalograms. Am J Orthod Dentofac Orthop.

[26] Damstra J, Slater JJRH, Fourie Z, Ren Y (2010). Reliability and the smallest detectable differences of lateral cephalometric measurements. Am J Orthod Dentofac Orthop.

[27] Koo TK, Li MY (2016). A guideline of selecting and reporting intraclass correlation coefficients for reliability research. J Chiropr Med.

[28] Gascho D, Philipp H, Flach PM (2018). Standardized medical image registration for radiological identification of decedents based on paranasal sinuses. J Forensic Leg Med.

[29] Jablonski NG, Shum BSF (1989). Identification of unknown human remains by comparison of antemortem and postmortem radiographs. Forensic Sci Int.

[30] Aydınlıoğlu A, Kavaklıı A, Erdem S (2003). Absence of frontal sinus in Turkish individuals. Yonsei Med J.

[31] Haglund WD (1992). Remains identification by frontal sinus radiographs. J Forensic Sci.

[32] Prossinger H, Bookstein FL (2003). Statistical estimators of frontal sinus cross section ontogeny from very noisy data. J Morphol.

[33] Christensen AM (2005). Assessing the variation in individual frontal sinus outlines. Am J Phys Anthropol.

